# Supplementary material for: Differences in Associations of Three Types of Alcoholic Beverages with Age-Related Cognitive Decline in Men
Source: Nutrients. 2024 Oct 30;16(21):3714. doi: 10.3390/nu16213714 (PMC11548026; doi:10.3390/nu16213714)
Supplement: Supplementary file 1 [file nutrients-16-03714-s001.zip › nutrients-3242964-supplementary.pdf]

Table S1. Associations between adult-life wine, beer and spirits consumption and IQ changes in men adjusted for additional variables including extreme binge drinking through adult life, other health behaviors, and morbidity (N=2456)

	**Model 2 + extreme binge drinking [a]**		**Model 2 + health behaviors [b]**		**Model 2 + morbidity [c]**	
	B (95% CI)	*p*-Value	B (95% CI)	*p*-Value	B (95% CI)	*p*-Value
Weekly units of wine						
0 units	Ref.	-	Ref.	-	Ref.	-
1–7 units	0.88 (-0.18;1.94)	0.102	1.05 (-0.02;2.12)	0.054	0.66 (-0.40;1.71)	0.223
8–14 units	1.64 (0.31;2.97)	0.016	1.51 (0.17;2.85)	0.028	1.06 (-0.27;2.38)	0.119
≥15 units	0.74 (-1.01;2.50)	0.406	0.30 (-1.46;2.06)	0.738	0.29 (-1.45;2.03)	0.744
Weekly units of beer						
0 units	Ref.	-	Ref.	-	Ref.	-
1–7 units	0.35 (-0.87;1.57)	0.577	0.23 (-1.00;1.46)	0.713	0.19 (-1.03;1.41)	0.760
8–14 units	-0.21 (-1.73;1.31)	0.797	-0.86 (-2.39;0.67)	0.269	-0.55 (-2.06;0.95)	0.472
≥15 units	0.17 (-1.55;1.89)	0.847	-1.42 (-3.09;0.24)	0.094	-0.09 (-1.77;1.58)	0.915
Weekly units of spirits						
0 units	Ref.	-	Ref.	-	Ref.	-
1–7 units	0.88 (1.45;1.62)	0.019	0.82 (0.08:1.56)	0.030	0.90 (0.17;1.63)	0.017
8–14 units	2.02 (-0.51;4.54)	0.117	1.17 (-1.37; 3.72)	0.366	1.93 (-0.57;4.43)	0.131
≥15 units	0.54 (-2.82;3.89)	0.753	-1.12 (-4.48;2.24)	0.514	1.09 (-1.77;1.58)	0.524

IQ = intelligence quotient calculated using Børge Priens Prøve test scores. Model 2: adjusted for consumption of other alcoholic beverages (i.e., mutual adjustment by including weekly units of wine, beer, and spirits in the same model), year of birth, age at follow-up, retest interval length, young adulthood IQ, years of education and personality. One unit of alcohol = 12 grams of pure alcohol. [a] Extreme binge drinking is defined as consuming 10 units of alcohol or more on the same occasion. [b] Health behaviors include pack-years of smoking, physical inactivity in leisure time, and number of years with weekly use of psychoactive drugs. [c] Morbidity includes alcohol-related hospital diagnoses, other mental disorder hospital diagnoses and charlson comorbidity index score.
